# How do carious root lesions develop after the end of professional preventive measures?—Preliminary findings of a randomized clinical trial

**DOI:** 10.1007/s10266-022-00706-8

**Published:** 2022-04-11

**Authors:** Deborah Kreher, Viktoria Korn, Thomas Meißner, Rainer Haak, Gerhard Schmalz, Dirk Ziebolz

**Affiliations:** Department of Cariology, Endodontology and Periodontology, University of Leipzig, Liebigstr. 12, 04103 Leipzig, Germany

**Keywords:** Root caries, QLF, Prevention, Non-invasive treatment, Varnish application

## Abstract

Aim of this randomized clinical trial was to assess the development of root caries lesions with and without (adjuvant) professional prevention treatment over 24 months. 20 participants with two or three non-cavitated root carious lesions were included (*n* = 52), whereby lesions were randomly assigned to one out of three groups depending on varnish application (CF: Cervitec F [*n* = 20], P: placebo [*n* = 20], DP: Duraphate [*n* = 12]). All lesions were assessed by quantitative light-induced fluorescence (QLF; QRayCam); following outcome parameters were analyzed: fluorescence loss (Δ*F* %), lesion volume (Δ*Q* %µm^2^) and bacterial activity (Δ*R* %). Professional tooth cleaning and adjuvant varnish application were performed at baseline, after 3, 6, and 9 months. A follow-up examination was performed 1 year after preventive care with varnish application 24 months after baseline. ∆*F* showed a significant time effect in CF (*p* = 0.03), which was not confirmed in post hoc analysis (*p* > 0.05). For P and DP, no time effect was detected (*p* > 0.05). ∆Q was significantly higher 12 months after baseline in CF (*p* = 0.02). In P, a significant time effect occurred (*p* = 0.01), without significant results in post hoc testing. ∆*R* showed higher values at baseline vs. 12 months in CF (*p* = 0.03) and 24 months compared to 12 months in DP (*p* = 0.02). Professional preventive treatment inhibited the progression of root caries lesions beyond their termination for 12 months, irrespective of an adjunctive varnish application. Preventive measures have a long-term effect on root carious lesions, even 1 year after their termination.

## Introduction

In the last decades, root caries has become a significant health problem; from 1997 to 2014, the prevalence has tripled [1]. Due to an increasing number of own teeth, patients often suffer from periodontitis and subsequently also from recession and exposed root surfaces [2, 3]. If prevalence remains equally, 16.5 million lesions will be present in 11.5 million people in the age group between 65 and 74 in 2030 [1]. Thus, non-invasive diagnostic and therapeutic procedures for root carious lesions will play an increasing role in daily dental practice.

Diagnosis and especially the treatment of root caries lesions is a major challenge [4]. Visual-tactile assessment is considered a standard method in clinical practice [5], but due to reliability problems of this process, the longitudinal observation is difficult [5, 6]. For this reason, additional diagnostic tools are desirable to provide further diagnostic information. These include light-based methods, for example, quantitative light-induced fluorescence (QLF). Previous studies show that QLF is a reproducible and valid method to detect root carious lesions in an early state [7, 8]. Moreover, reproducible monitoring is also possible [9].

Similar to the different diagnostic approaches, various treatment strategies are also available; these include non-invasive therapy, e.g., with varnish application, while advanced lesions often require restorations [10]. However, restorations placed in this area often have a poor long-term success rate [4, 10, 11]. Therefore, an early diagnosis and non-invasive assessment of root caries, alongside with the opportunity to monitor lesions, is needed [4].

There is no gold standard therapy for the non-invasive treatment of root caries [12]. Nevertheless, it was found that daily use of toothpaste containing 5000 ppm fluoride seems to be highly effective against active root caries [12]. Furthermore, professionally applied CHX varnish shows decreasing the number of root caries. Moreover, the daily use of dentifrice containing 1.5% arginine plus 1450 ppm fluoride inactivates more lesions than a fluoridated toothpaste alone [12]. A previous study of this working group has shown that preventive care positively influences demineralized root surfaces over 12 months. Thereby, it could be demonstrated that topical adjuvant varnish application (e.g., based on the combination of CHX and fluoride 1.450 ppm) can help to arrest or even remineralize carious root lesions [9]. Regardless of those findings, long-term effects are not known, yet. Particularly, it is unclear what happens if preventive measures are not continued; therefore, information on the development of root caries lesions after preventive therapy is not available until now.

Therefore, this current randomized clinical trial aimed at the assessment of the development of root caries lesions over a period of 24 months, including a 12-month-lasting period of application of non-invasive professional preventive measures with different adjuvant varnish application, following 12 months without any adjuvant (study-related) professional preventive measure based on QLF measurement. Thereby, it should be assessed, how lesions would develop 1 year after termination of preventive therapy. It was therefore hypothesized that non-invasive preventive measures lead to arrest or remineralization of the lesions, while a progression of the lesion 12 months after the termination of preventive measures is expected.

## Materials and methods

### Study design

**Fig. 1:**
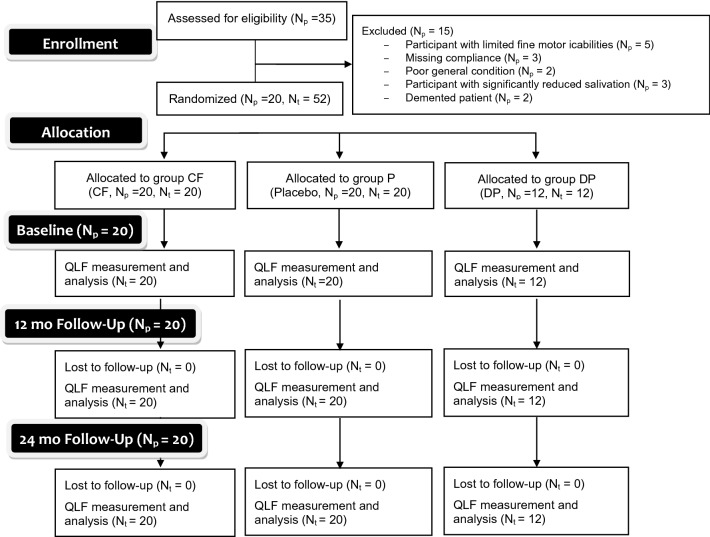
Participant flow through the randomized clinical trial (RCT) according to the CONSORT guidelines

The current study was performed based on a previous clinical trial of this working group [9]. The patient cohort from the previous trial was included in this 24-month follow-up by specific in- and exclusion criteria (see below). The full prospective, double-blind, placebo-controlled, three-armed randomized controlled trial (RCT) has been reviewed and approved by the ethics committee of the medical faculty of the University of Leipzig (429/16-ek). Furthermore, the study was registered in the "WHO international clinical trials registry platform" in the German clinical trial register (DRKS; No: DRKS00011217). All procedures were performed in full accordance with the Declaration of Helsinki. The participating patients were informed verbally and in writing about the study and gave their written informed consent. The flowchart according to CONSORT guidelines is given in Fig. 1.

### Participants

A minimum sample size of 18 teeth for each group was determined based on a two-armed parallel study design to detect differences with at least 80% statistical power. A significance level of 5% irrespective of a normal distribution was considered.

The inclusion and exclusion criteria were equal as in the previous study [9], i.e., inclusion criteria:

- age between 60 and 79 years
- two or three exposed, non-adjacent root surfaces with initial (non-cavitated) carious lesions on permanent anterior teeth or premolars (maxilla or mandible)
- completion of the whole 24-month follow-up

exclusion criteria:

- limited motoric abilities affecting oral health procedures
- poor general health
- reduced salivary flow
- dementia
- immunosuppression
- malignancies
- Hepatitis A, B, C, TBC, HIV
- addiction (alcohol dependence)
- allergy against ingredients of used agents

### Test material and group allocation

Three groups were examined in this current study. First, an ammonium fluoride-/chlorhexidine- (CHX)/cetylpyridinium chloride (CPC) containing varnish (CF group: Cervitec F; Ivoclar Vivadent AG, Schaan, Lichtenstein), second, a placebo varnish (P group: Ivoclar Vivadent AG) based on the essential composition of Cervitec F, excluding ammonium fluoride, CHX and CPC and third, in case of presence of a third tooth with a respective lesion, a high fluoride varnish (DP group: Duraphat; Colgate Oral Pharmaceutical, Inc, Canton MA, USA). The detailed composition of the tested materials can be assessed in the previously published study [9]. The included teeth were randomly assigned to one out of the three groups. An independent person performed the whole randomization and allocation process.

### QLF assessment and outcome parameter

The primary outcome parameter of the current study were QLF analyzed (QRayCam v.1.00, serial no.: 15090005, Software C3 v 1.26 Inspektor Research Systems, Amsterdam, Netherlands), including fluorescence loss/ demineralization states (Δ*F*, %), lesion volume/area (Δ*Q*, mm^2^ × %) and increase of red fluorescence (Δ*R*, %). Therefore, the QLF method was applied as described previously [9].

### Study flow

**Fig. 2:**
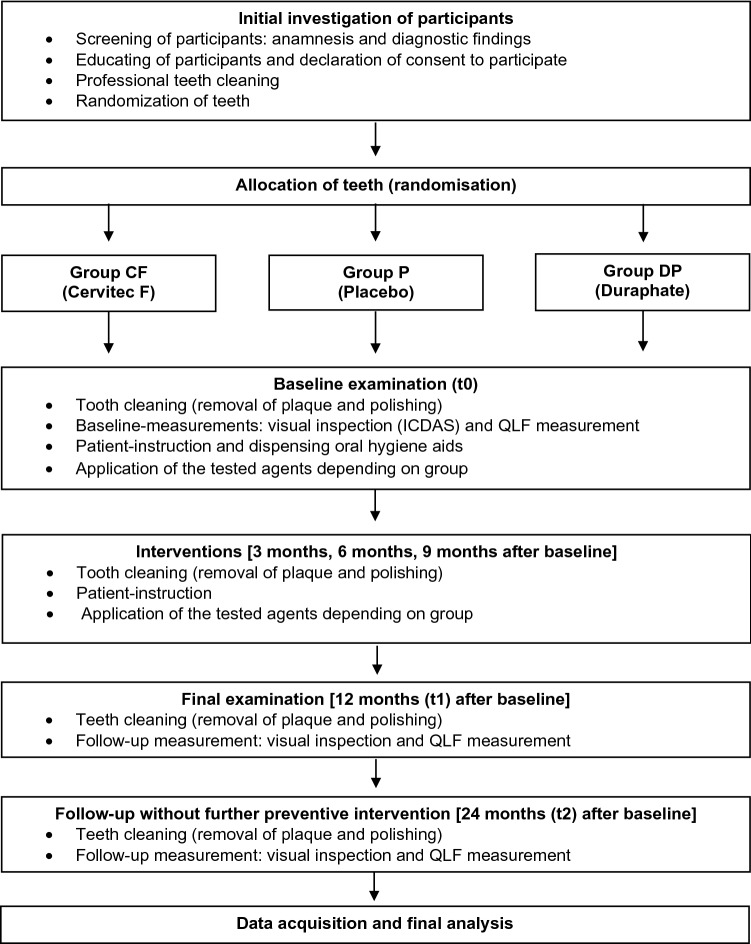
Workflow

All investigations were carried out between April 2017 to August 2019. The flow of the current study is displayed in Fig. 2. Following an initial check of the eligibility criteria, patients were informed verbally and in writing and provided written informed consent. A professional tooth cleaning was applied to all included participants at baseline to ensure equal starting conditions. One experienced, calibrated (kappa > 0.8) and blinded dentist performed all examinations.

At baseline, the first QLF measurement was performed. Subsequently, the plaque was removed without polishing paste (Prophy Angle Lavender Soft Cup, LOT: 20,170,109, Dentsply Sirona, York, USA), followed by visual inspection (score 1 and 2 of ICDAS). All participants received oral hygiene aids for dental home care (toothbrush: SUNSTAR GUM ActiVital, Sunstar Deutschland GmbH Kriftel, Germany; toothpaste: Dentagard, Colgate Oral Pharmaceutical, Inc, Canton MA, USA; renewed every three months) and an oral hygiene instruction (brushing twice daily for 2 min.). Patients were prohibited from using any other oral hygiene products or chemical devices besides the received ones. Two or three different agents were applied according to group allocation after the baseline examination and repeated 3-monthly for the first year (3, 6, and 9 months after baseline). At each appointment for varnish application, patients received professional preventive measures, including professional tooth cleaning and reinstruction for oral hygiene as well as respective varnish application. In the second year of the observational period, no professional preventive measures, i.e., no adjuvant (study-related) varnish applications, tooth cleaning, or other measures were performed. However, whether the patients had received any respective measures elsewhere was not checked.

The QLF measurement was performed on the test teeth according to baseline examination. A calibrated and blinded investigator assessed all QLF images under standardized conditions (artificial light, no window, air conditioner set to 23 °C). After the baseline QLF examination, further examinations were performed after 12 (t1) and 24 months (t2).

### Statistical analysis

SPSS for Windows, Version 24.0 (SPSS Inc., U.S.A.) was applied for statistical analysis. Shapiro–Wilk-test was applied to test for normal distribution of the sample. Levene-test was used to test for homogeneity of variance and to show an appropriate similarity in the allocation of the samples, which allows univariate analysis. The general linear model analyzed more than two dependent, normally distributed samples. Non-normal distributed samples were tested by Friedman-test. Post hoc testing using Least Significant Difference as well as Bonferroni test was performed in case of statistical significance (significance level: *p* ≤ 0.05).

## Results

### Patients

Twenty participants could finally be included in the current study. Within these patients, a total of 52 teeth with cervical lesions (CF: *n* = 20, P: *n* = 20, and DP: *n* = 12) were evaluated. All baseline parameters, including gender, age, ICDAS code, and salivary parameters, were comparable between groups (*p* > 0.05; Table 1). Out of the included teeth, 15 teeth were in the anterior region of the maxilla, while five teeth were in the posterior region of the maxilla. Furthermore, 21 teeth were examined in the anterior region of the lower jaw and 11 teeth in the posterior region of the mandible (Table 2).

**Table 1:** Patients characteristics

	Total	Group CF	Group P	Group DP	*p*-value
Number of teeth (*n* [%])	52 (100)	20 (38)	20 (38)	12 (24)	–
Gender (*n* [%])					
Female	24 (46)	9 (45)	9 (45)	6 (50)	0.88
Male	28 (54)	11 (55)	11 (55)	6 (50)	
Age in years (mv ± sd)	66.10 ± 9.71	66.15 ± 9.79	66.15 ± 9.79	65.92 ± 9.44	0.98
ICDAS II Score (*n* [%])					
Score 1	38 (73)	15 (75)	14 (70)	9 (75)	0.82
Score 2	14 (27)	5 (25)	6 (30)	3 (25)	
Salivary flow rate (*n* = 53; ml/5 min; (mv ± sd)					
Un-stimulated	0.93 ± 0.75	0.92 ± 0.75	0.92 ± 0.75	0.97 ± 0.76	0.96
Stimulated	5.28 ± 3.01	5.23 ± 3.09	5.23 ± 3.09	5.41 ± 3.07	0.99
Reduced salivary flow (*n* [%])	0	0	0	0	–
Salivary buffer capacity (*n* = 53; *n* [%])					
Low	3 (6)	1 (5)	1 (6)	1 (8)	0.87
Medium	11 (21)	4 (24)	4 (24)	3 (25)	
High	33 (63)	12 (71)	13 (72)	8 (67)	

*CRT* buffer Test, Ivoclar Vivadent, Schaan Liechtenstein, *mv* mean value, *sd* standard deviation]

**Table 2:** Distribution of included teeth between groups

	Total (*n* = 52)	Group CF (*n* = 20)	group P (*n* = 20)	Group DP (*n* = 12)
Anterior maxilla	15	8	6	1
Premolar maxilla	5	0	3	2
Anterior mandible	21	7	8	6
Premolar mandible	11	5	3	3

### QLF results

All findings of QLF analysis are shown in Tables 3 and 4.

**Table 3:** Results for Δ*F*, Δ*Q*, and ∆*R* depending on the time of examination for the total cohort (mean [SD])

	Total (*n* = 52)
Δ*F* (%)	
Baseline	– 12.92 (10.48)
T1	– 10.77 (9.93)
T2	– 15.01 (14.27)
*p*-value	** < 0.01**
Baseline vs. 12 months	** < 0.01**
Baseline vs. 24 months	0.99
12 months vs. 24 months	**0.01**
Δ*Q* (%µm^2^)	
Baseline	– 13,453.42 (22,860.31)
T1	– 7787.21 (16,163.49)
T2	– 14,577.82 (31,398.83)
*p*-value	< 0.01
Baseline vs. 12 months	** < 0.01**
Baseline vs. 24 months	**0.02**
12 months vs. 24 months	0.72
Δ*R* (%)	
Baseline	25.92 (35.15)
T1	19.01 (23.09)
T2	34.38 (39.66)
*p*-value	** < 0.01**
Baseline vs. 12 months	** < 0.01**
Baseline vs. 24 months	0.99
12 months vs. 24 months	** < 0.01**

Significant findigs are highlighted in bold (**p** < 0.05)

**Table 4:** Results for Δ*F*, Δ*Q*, and ∆*R* depending on the time of examination for groups (mean [SD])

	Group CF	Group P	group DP
Δ*F* (%)			
Baseline	– 12.68 (8.95)	– 12.07 (8.78)	– 14.70 (15.30)
T1	– 9.68 (8.64)	– 10.56 (8.10)	– 12.92 (14.43)
T2	– 13.56 (11.92)	– 13.65 (13.51)	– 19.67 (18.80)
*p*-value	**0.03**	0.05	0.09
Baseline vs. 12 months	0.53	–	–
Baseline vs. 24 months	0.99	–	–
12 months vs. 24 months	0.17	–	–
Δ*Q* (%µm^2^)			
Baseline	– 10,369.53 (14,495.52)	– 13,338.16 (20,007.63)	– 18,785.35 (36,335.02)
T1	– 5157.79 (8023.49)	– 7163.67 (9705.18)	– 13,208.81 (29,867.32)
T2	– 8473.74 (11,023.93)	– 11,530.35 (18,102.41)	– 29,830.41 (58,792.63)
*p*-value	**0.02**	**0.01**	0.15
Baseline vs. 12 months	**0.02**	0.05	**– **
Baseline vs. 24 months	0.34	0.05	**– **
12 months vs. 24 months	0.81	0.99	**– **
Δ*R* (%)			
Baseline	22.14 (21.77)	22.44 (21.10)	38.01 (62.72)
T1	16.62 (19.27)	19.00 (18.23)	23.00 (35.07)
T2	27.08 (24.34)	32.63 (40.69)	49.48 (55.30)
*p*-value	** < 0.01**	0.16	**0.01**
Baseline vs. 12 months	**0.03**	–	0.31
Baseline vs. 24 months	0.99	–	0.92
12 months vs. 24 months	0.10	–	**0.02**

Significant findings are highlighted in bold (*p* < 0.05)

#### Fluorescence loss (Δ*F*, %)

Regarding the parameter Δ*F*, there was a significant time effect in the total sample (*p* < 0.01), whereby significant differences were found between baseline vs. 12 months (*p* < 0.01) and 12 months vs. 24 months (*p* = 0.01, Table 3).

In group comparison a significant time effect was only detected in CF (*p* = 0.03), while post hoc analysis did not show any significant difference (*p* > 0.05). Moreover, the P trended to show a time effect (*p* = 0.05, Table 4).

#### Lesion volume / area (Δ*Q*, mm^2^ × %)

For Δ*Q*, a significant time effect was shown in the total group (*p* < 0.01). Post hoc analysis revealed significant differences between baseline vs. 12 months (*p* < 0.01) and baseline vs. 24 months (*p* = 0.02, Table 3). In group comparison, there was a significant time effect in CF (*p* = 0.02). Post hoc analysis shows a significant difference between baseline vs. 12 months (− 10,369.53 ± 14,495.52 vs. − 5157.79 ± 8023.49, *p* = 0.02). In P, a significant time effect could be confirmed, too (*p* = 0.01). However, post hoc testing did only reveal a trend between baseline and 12 months as well as between baseline and 24 months (*p* = 0.05, Table 4).

#### Red fluorescence (Δ*R*, %)

Regarding Δ*R*, a significant time effect was shown in the total sample, independent of the intervention group (*p* < 0.01). Thereby, the differences reached significance between baseline vs. 12 months (*p* < 0.01) and 12 months vs. 24 months (*p* < 0.01, Table 3). Comparing the three groups, there was a significant time effect in CF (*p* < 0.01), whereby post hoc analysis showed a difference between baseline vs. 12 months (22.14 ± 21.77 vs. 16.62 ± 19.27, *p* = 0.03). Furthermore, there was a significant time effect in DP (*p* = 0.01), while post hoc testing confirmed a significance between 12 and 24 months (23.00 ± 35.07 vs. 49.48 ± 55.30, *p* = 0.02, Table 4).

## Discussion

Within the total sample, including 52 teeth, there was a significant time effect in all QLF parameters during the study period; thereby, only Δ*Q* values differed between baseline and 24 months (i.e., 12 months after termination of professional preventive measures). Summarising the main results of comparing the three groups, no significant differences were found in all QLF parameters at the last examination date after 24 months compared to baseline and the values fell back to the baseline level. Only within CF group, Δ*Q* and Δ*R* were significantly reduced after 12 months of 3-monthly prevention and varnish application. Differences between 12 and 24 months, i.e., 1 year after preventive measures and varnish application stopped, were only found for ΔR in DP group.

The existing literature shows that no gold standard is available in treating root carious lesions [4, 10]. Basically, oral hygiene is a significant predictor for the risk of developing root caries [13]. Therefore, managing personal oral health measures appear one crucial therapeutic approach. Besides, it has been known for many years that professional preventive measures, including professional tooth cleaning, significantly reduce the incidence and activity of (root) caries [14]. Nearly 30 years ago, Emilson et al. showed that a 12-month preventive program led to reduced activity of carious root surfaces [15]. Therefore, a positive effect of preventive measures in general, as observed in the current study, appears plausible and not surprising.

This current RCT examined three adjuvant intervention approaches, including CHX/CPC combined with fluoride varnish application, high fluoride-containing varnish, and a placebo group. Some studies have shown that high fluoride dentifrice with 5000 ppm fluoride seems to be very efficient to arrest root surface caries [10, 16, 17]. Another systematic review concluded that 38% silver diamine fluoride (SDF) solution combined with dental health education would be the most effective intervention against root caries [18]. For oral hygiene at home, the daily application of 0.2% sodium fluoride (NaF) seems to be the most effective [18]. Moreover, CHX alone or the combination of fluoride and CHX was reported to be a highly effective prevention measure for root caries, respectively [12, 19]. Thereby, CHX might be especially beneficial if regular oral hygiene measures and tooth cleaning were not possible [19], which does not comply with the current study's design. Current in vitro studies reported the combination of CHX and fluoride as the most successful measure to arrest root caries and reduce respective microbiological load [20, 21]. However, the current study's findings are a little surprising against the background of beneficial effects of fluoride alone or combined with CHX/CPC. The previous study of this working group, which relayed on the same patient cohort as the current study, has investigated the effect of different adjuvant varnish applications with a follow-up period of 12 months. It could be shown that both Cervitec F and Duraphat positively affected carious root lesions. However, a positive effect was seen in the placebo group as well [9]. This could allow the conclusion that any kind of prevention leads to the improvement of root caries, irrespective of the form of intervention or adjuvant application, respectively; thus, professional tooth cleaning seems to have a more significant effect than adjuvant varnishes. This appears to confirm the findings of Axelsson et al. and Emilson et al. accordingly [14, 15].

However, the most interesting question of the current study was: what happens after adjuvant (study-related) professional preventive measures and varnish application? Interestingly, no difference between baseline and after 24 months (i.e., 12 months with and 12 months without preventive measures) was found across groups, which was also evident for the total sample (*n* = 52). Thereby, the similarity in Δ*F* between baseline and 24 months indicates that lesion depth did not increase, which argues for a arrest of the respective root caries lesions. Therefore, in the current study, the investigators could show that the baseline situation is restored after prevention stopped. This fact seems to be independent of the type of varnish. Initially, it was hypothesized that non-invasive preventive measures lead to arrest or remineralization of the lesions, while a progression of the lesion 12 months after the termination of preventive measures is expected. This hypothesis was not confirmed, as there was no significant progression but rather a restoration of the baseline situation. One potential explanation could be a methodological concern of the current study. This study exclusively applied the QLF method. Another study of this working group has shown that the percentage of fluorescence loss (Δ*F*) is a predictor for lesion depth in root carious lesions. Hence the method allows a quantitative evaluation of these lesion depths, especially of non-cavitated root surface caries [8]. Considering the results for ΔF in the current study, a significant reduction or increase of lesion depth is not observed at any time point or in any group (see Table 3). This would rather argue for an arrest of the lesion instead of changes in mineralization.

Two other parameters were assessed by QLF; Δ*Q* reflects the percentage of fluorescence loss related to lesion volume (i.e., the product of Δ*F* and the area of the lesions in mm^2^) and ΔR, which shows the relationship between red and green fluorescence depending on the presence of porphyrins [22]. Those parameters were improved after 12 months in the CF group, which argues for a reduced lesion volume and activity of the root caries lesions. However, after 24 months, no differences with regard to 12 months or baseline were confirmed. Only in the DP group, such an effect was found; based on the low sample size and the spreading of results, the interpretation of these findings is unclear. ΔQ has been mentioned in the context of remineralization of carious lesions in various studies [23, 25]. Thereby, it has been found that Δ*Q* of white spot lesions decreased after continuous chewing of chewing gum [25]. Moreover, ΔQ was reduced on buccal carious lesions after non-invasive treatment with fluoride-containing dentifrice [23]. While these findings were in enamel, one study found Δ*Q* to be reduced after fluoride application on root caries, indicating the ability of this parameter to quantify remineralization and detect arrest of the lesions [24].

Thus, the fact that both Δ*Q* and Δ*F* were not significantly different between baseline and 24 months in the current study could indicate that the lesions were arrested beyond the termination of preventive measures. Additionally, the activity of the carious lesions could be reduced with a long-term effect for the same period. However, this remains somewhat speculative and is insufficiently supported by the current study's findings. Professional preventive measures have a positive effect beyond the point of termination regardless of this.

The strengths of this study are as follows: It was designed as an RCT with patients divided into three groups (CF, P, DP) and subjected to standardized examination. A follow-up period was defined for 2 years, which is a further strength. Moreover, QLF as a non-invasive method has been validated for root caries assessment concerning Δ*F* [8]. Nevertheless, there are some limitations of this study, too. Due to the small sample size, especially in DP, these results should only be considered preliminary caused by their limited power. Therefore, the whole sample has been examined, too, aiming in achieving a conclusion, which is as reliable as possible. However and regardless, the limited power must be recognized for the interpretation of the current study´s findings, which need validation in further studies with larger sample size. In this context, three patients were excluded because of “missing compliance”. As mentioned in methods section, DP was only applied if patients had a third tooth with root caries. Unfortunately, all of those excluded individuals had such a third lesion, resulting in the problem that the initially smallest DP group was even smaller and below the power threshold. This is a potential bias, affecting the study outcome and the ability to draw robust conclusions.

Furthermore, it is unclear what exactly happens to the lesions (remineralization, demineralization, arrest), so the conclusions remain partly speculative. After the intervention period (between 12 and 24 months after baseline), patients might have used different oral hygiene measures or visited a dentist, probably affecting the current findings. Therefore, the current study can only support conclusions related to the professional prevention measures applied here. Also, this study did not consider patient-specific data (general diseases, medication, and nutritional behavior). Especially disease- or medication-related xerostomia alongside with nutritional habits (e.g., sugar consumption) could affect the root caries progression in the current sample [26], what might also influence the current results. Changes in general health, mental state or motor skills that have not been explicitly recorded may have occurred during the study period. It is known that especially elderly individuals show manual dexterity and cognitive impairment, which affects their ability to perform oral hygiene measures, increasing their need for professional prevention in a higher frequency [27]. Future studies should investigate the effect of preventive care with larger group size, also considering patient-specific parameters. Prevention strategies should primarily include regular professional tooth cleaning and monitoring as the most important intervention. A higher frequency of follow-up measurements (e.g., 3-monthly) and a longer follow-up might be recommendable to gain more insight into what happens after the termination of professional preventive measures.

## Conclusion

Within the limitations of this preliminary study, professional preventive measures have an effect on root caries lesions beyond their termination over a period of 12 months. This effect is mainly irrespective of an adjunctive varnish application with fluoride or a combination of fluoride and CHX. Future studies need to show the exact processes within the lesion to derive clinical implications.
